# Serological Responses and Biomarker Evaluation in Mice and Pigs Exposed to Tsetse Fly Bites

**DOI:** 10.1371/journal.pntd.0002911

**Published:** 2014-05-22

**Authors:** Guy Caljon, Reta Duguma, Reginald De Deken, Stijn Schauvliege, Frank Gasthuys, Luc Duchateau, Jan Van Den Abbeele

**Affiliations:** 1 Unit of Veterinary Protozoology, Department of Biomedical Sciences, Institute of Tropical Medicine Antwerp (ITM), Antwerp, Belgium; 2 Unit of Cellular and Molecular Immunology, Vrije Universiteit Brussel (VUB), Brussels, Belgium; 3 Laboratory of Myeloid Cell Immunology, Vlaams Instituut voor Biotechnologie (VIB), Brussels, Belgium; 4 Faculty of Veterinary Medicine, Addis Ababa University, Debre Zeit, Ethiopia; 5 Unit Veterinary Entomology, Department of Biomedical Sciences, Institute of Tropical Medicine Antwerp (ITM), Antwerp, Belgium; 6 Department of Surgery and Anaesthesia of Domestic Animals, Faculty of Veterinary Medicine, Ghent University (UGent), Ghent, Belgium; 7 Department of Comparative Physiology and Biometrics, Faculty of Veterinary Medicine, Ghent University (UGent), Ghent, Belgium; 8 Laboratory of Zoophysiology, Department of Physiology, Ghent University (UGent), Ghent, Belgium; IRD/CIRDES, Burkina Faso

## Abstract

**Background:**

Tsetse flies are obligate blood-feeding insects that transmit African trypanosomes responsible for human sleeping sickness and *nagana* in livestock. The tsetse salivary proteome contains a highly immunogenic family of the endonuclease-like Tsal proteins. In this study, a recombinant version of Tsal1 (rTsal1) was evaluated in an indirect ELISA to quantify the contact with total *Glossina morsitans morsitans* saliva, and thus the tsetse fly bite exposure.

**Methodology/Principal Findings:**

Mice and pigs were experimentally exposed to different *G. m. morsitans* exposure regimens, followed by a long-term follow-up of the specific antibody responses against total tsetse fly saliva and rTsal1. In mice, a single tsetse fly bite was sufficient to induce detectable IgG antibody responses with an estimated half-life of 36–40 days. Specific antibody responses could be detected for more than a year after initial exposure, and a single bite was sufficient to boost anti-saliva immunity. Also, plasmas collected from tsetse-exposed pigs displayed increased anti-rTsal1 and anti-saliva IgG levels that correlated with the exposure intensity. A strong correlation between the detection of anti-rTsal1 and anti-saliva responses was recorded. The ELISA test performance and intra-laboratory repeatability was adequate in the two tested animal models. Cross-reactivity of the mouse IgGs induced by exposure to different *Glossina* species (*G. m. morsitans*, *G. pallidipes*, *G. palpalis gambiensis* and *G. fuscipes*) and other hematophagous insects (*Stomoxys calcitrans* and *Tabanus yao*) was evaluated.

**Conclusion:**

This study illustrates the potential use of rTsal1 from *G. m. morsitans* as a sensitive biomarker of exposure to a broad range of *Glossina* species. We propose that the detection of anti-rTsal1 IgGs could be a promising serological indicator of tsetse fly presence that will be a valuable tool to monitor the impact of tsetse control efforts on the African continent.

## Author Summary

Salivary proteins of hematophagous disease vectors represent potential biomarkers of exposure and could be used in serological assays that are complementary to entomological surveys. We illustrate that a recombinant version of the highly immunogenic Tsal1 protein of the savannah tsetse fly (*Glossina morsitans morsitans*) is a sensitive immunological probe to detect contact with tsetse flies. Experimental exposure of mice and pigs to different regimens of tsetse fly bites combined with serological testing revealed that rTsal1 is a sensitive indicator that can differentiate the various degrees of exposure of animals. Tsetse-induced antibodies persisted relatively long, and an efficient boosting of immunity was observed upon re-exposure. Recombinant Tsal1 is a promising candidate to detect contact with various tsetse species, which would enable screening of populations or herds for exposure to tsetse flies in various areas on the African continent. This exposure indicator could be a valuable tool to monitor the impact of vector control programs and to detect re-invasion of cleared areas by tsetse flies.

## Introduction

Tsetse flies (*Glossina* spp.) are notorious transmitters of trypanosome parasites responsible for Human and Animal African Trypanosomiasis (HAT and AAT). Since 2009, the annual number of reported cases of HAT has dropped below 10000 (www.who.int; [1]) with the prospect and challenge of entering into the elimination phase of HAT in the near future [2], [3]. Additionally, some 46 million cattle in sub-Saharan Africa are estimated to be at risk of contracting AAT making deep inroads in the socio-economical development of this continent [4]. Beside active HAT case detection and treatment of humans as well as prophylactic and curative treatment of animals with trypanocidal drugs, tsetse vector control represents an important component of trypanosomiasis control, which is mainly based on the use of insecticides through the sequential aerosol spraying technique (SAT), ground spraying, insecticide-treated targets or insecticide-treated animals [reviewed in [5], [6], [7]]. After a successful campaign as part of an area-wide integrated pest management on Unguja island (Zanzibar) [8], the sterile insect technique has been added to the vector control arsenal, with ongoing activities in Ethiopia, Senegal and Burkina Faso [9] under the auspices of the Pan African Tsetse and Trypanosomosis Eradication Campaign (PATTEC). However, beside laborious conventional entomological surveys, no sensitive rapid tests are yet available to provide a semi-quantitative measure of the evolution of tsetse fly densities in areas subjected to tsetse control interventions. Indeed, easy-to-use monitoring of tsetse fly exposure on a regular basis would be a highly valuable tool in the follow-up of the efficacy of the applied and/or ongoing tsetse fly control activities.

The obligatory blood feeding tsetse flies are the cyclical insect vectors of HAT and a majority of AAT infections are initiated by the bite of an infected tsetse fly. Although it can be assumed that all tsetse fly species could act as vector, a number of *Glossina* species of the Palpalis group (e.g. *G. palpalis* spp., *G. fuscipes* spp., *G. tachinoides*) and the Morsitans group (e.g. *G. morsitans* spp., *G. pallidipes*, *G. swynnertoni*) are implicated as major vectors for HAT and animal trypanosomiasis. Given that only a limited percentage of these tsetse flies acquire an infection with trypanosomes, vertebrate hosts living in the tsetse fly belt are predominantly exposed to the bites of uninfected flies. It has been demonstrated for a number of hematophagous insects that salivary proteins induce humoral immune responses that could represent attractive sero-epidemiological markers of exposure (reviewed in [10]). The saliva of *Glossina morsitans morsitans* tsetse flies was documented to contain over 200 protein constituents [11] from which some are implicated in manipulating the vertebrate hemostatic and inflammatory reactions [12], [13], [14]. In *G. m. morsitans* saliva, the most abundant proteins were shown to be highly immunogenic and to correspond to the 43–45 kDa tsetse salivary gland (Tsal) protein family [15]. The physiological role of these proteins remains elusive, but biochemical characterization revealed that they are nucleic acid binding proteins with low endonuclease activity [16]. Immunoglobulin responses to tsetse fly saliva have been detected in humans living in Uganda [15], Democratic Republic of Congo [17], [18] and Guinea [19]. Also cattle experimentally exposed to tsetse fly bites displayed elevated levels of anti-saliva antibodies [20]. Immunoblotting studies using the immune plasmas have shown that salivary proteins of several tsetse fly species are recognized by the circulating antibodies [15], [18], [19]. The highly abundant Tsal proteins were commonly recognized by the human plasmas and an indirect ELISA using recombinant Tsal1 and Tsal2 proteins as antigens was clearly able to differentiate the tsetse-exposed Ugandan plasmas from control plasmas [15]. Recently, a peptide (amino acids 18–43) derived from the *G. m. morsitans* adenosine deaminase-related TSGF1 protein was evaluated using a panel of human plasmas from West Africa, revealing that obtained ELISA signals correlated with the anticipated levels of tsetse exposure of the tested populations [21]. Allergic and anaphylactic reactions against tsetse fly bites have also been reported, in which IgE antibodies directed against an Antigen5-related salivary allergen are implicated [22], [23], [24]. Efforts to develop a serological test based on a TAg5-derived peptide were not yet successful [21].

We here provide experimental evidence that anti-rTsal1 and anti-*G. m. morsitans* saliva antibodies can mark exposure of mice and pigs to tsetse flies. Although the anti-tsetse saliva ELISA exhibits a better average test performance, we propose that a serological assay based on the individual recombinant Tsal1 protein could be an alternative to assess the exposure of populations or herds to tsetse fly challenge and hence could be used for tsetse fly epidemiological studies, for prioritizing tsetse fly control, for monitoring and evaluating tsetse fly control schemes and for risk assessment of trypanosome transmission in endemic regions.

## Methods

### Ethics statement

The experiments, maintenance and care of animals complied with the guidelines of the European Convention for the Protection of Vertebrate Animals used for Experimental and other Scientific Purposes (CETS n° 123). Rodent care and experimental procedures were performed under approval from the Animal Ethical Committee of the Institute of Tropical Medicine (Permit Nrs. PAR013-MC-M-Tryp and PAR014-MC-K-Tryp). Breeding and experimental work with tsetse flies was approved by the Scientific Institute Public Health department Biosafety and Biotechnology (SBB 219.2007/1410). Pig experiments were approved by the ITM Animal Ethical Committee (Permit Nr. PAR-021) and the Ethical Committee of the Faculty of Veterinary Medicine of Ghent University (Permit Nr. EC2010/030) and were performed in the Ghent university stables under the supervision of a veterinary doctor.

### Salivary antigens and recombinant proteins

Saliva of *Glossina m. morsitans* from the ITMA tsetse fly colony was collected as outflow from the salivary glands as described elsewhere [16]. Saliva of *Tabanus yao* was kindly provided by Prof. Ren Lai (Kunming Institute of Zoology, Yunnan, China), *Stomoxys calcitrans* saliva was harvested from flies received from Dr. Christopher J. Geden (United States Department of Agriculture, Gainesville, US). Recombinant Tsal1 was purified as described elsewhere from inclusion bodies of IPTG-induced Top10F' *Escherichia coli* host cells harboring a pQE60:Tsal1 plasmid [15]. Tsal1 was resolubilized in 6M guanidinium hydrochloride, enriched by Ni-NTA affinity chromatography (Qiagen) and further purified on a Superdex 200 size exclusion column connected to an Akta Explorer (GE Healthcare) in 6 M ureum 50 mM Tris pH 8.0 and 600 mM NaCl. Protein concentrations were determined using Nanodrop spectrophotometry and samples were stored in aliquots at −20°C. *E. coli* soluble extract was prepared as an additive for the porcine ELISA assay diluent as described for other porcine assays [25]. The extract was made from a 1L overnight culture of Top10F' *E. coli* in Terrific broth. The bacterial pellet was resuspended in 12 ml PBS supplemented with a Complete protease inhibitor cocktail tablet (Roche), followed by 5 rounds of 1 minute sonication. The soluble fraction was harvested as the supernatant after 30 minutes centrifugation at 20.000× *g*. *E. coli* soluble extract was stored in aliquots at −20°C.

### Experimental study animals and immunization

Groups of eight female outbred mice (NMRI, Charles River) were subjected to different intensities of exposure to *G. m. morsitans* bites, followed by regular blood sampling and evaluation of the antibody responses in ELISA: (*i*) once exposed to a single fly, (*ii*) once exposed to 10 flies, (*iii*) 3 times per week for 3 weeks exposed to a single fly, (*iv*) 3 times per week for 3 weeks exposed to 10 flies and (*v*) not exposed to tsetse fly bites. One mouse that underwent multiple exposures to a single fly succumbed by day 28 after initiation of exposure. Plasmas were collected over a period of 390 days. After this >1 year non-exposure period, six mice that were exposed to single or multiple (10) flies were selected based on their physical appearance and behavior and were re-exposed to a single *G. m. morsitans* tsetse fly followed by weekly plasma collection for up to 42 days after re-challenge. Two mice that were previously exposed to the multiple fly bites succumbed within 4 weeks after the boosting.

A *Glossina* species cross-reactivity study was performed by exposing five mice (OF1, Charles River) per group every 3 days for 6 weeks to 10 flies of either *G. m. morsitans*, *G. p. gambiensis*, *G. pallidipes* (kindly provided by Peter Takac, Institute of Zoology, Bratislava, Slovakia) or *G. f. fuscipes* (kindly provided by the International Centre of Insect Physiology and Ecology, Mbita Point, Kenya). Immune plasma was harvested 10 days after the last exposure. Five non-exposed mice served as negative controls. A cross-reactivity study between salivary antigens of hematophagous insects was performed by intrapinna exposure of 6 OF1 mice per group at 3-weekly interval to decreasing amounts of saliva (5, 2, 1 and 1 µg) harvested from *Glossina morsitans*, *Stomoxys calcitrans* and *Tabanus yao*. Plasma samples were collected 10 days after the last immunization.

A total of 11 female pigs (Seghers, Belgium), hybrids of Belgian Landrace, Large White and a specific company line were used for experimental exposure to three different *G. m. morsitans* exposure regimens: (*i*) 5 pigs were weekly exposed for 7 weeks to 30 tsetse flies (high exposure), (*ii*) 5 pigs were exposed 2-weekly for 6 weeks to 3 tsetse flies (low exposure, 1 pig died at day 7) and (*iii*) 1 pig was not exposed to tsetse (negative control). The experimental pigs were 6 weeks old at the start of the experiment following a 4-days acclimatisation period in the university stables (Faculty of Veterinary Medicine, UGhent). For anaesthesia of the pigs, intramuscular injection of midazolam (0.5 mg/kg), ketamine (10 mg/kg) and morphine (0.1 mg/kg) was used. Blood was collected every week for 11 successive weeks from the jugular vein using Vacutainer EDTA tubes (BD) a few minutes after the pigs had been anesthetised and prior to exposure to the tsetse fly bites. After blood collection, the blood was centrifuged at 3000 rpm for 15 minutes and plasma stored at −20°C. Due to the unexpected death of 1 pig, no tsetse challenge and blood sampling was performed on day 7 for all animals.

Due to limited housing capacity in the university stables, only two pigs from the low exposure group could be kept for an additional 2-month period of non-exposure, followed by a re-challenge by the bites of 10 *G. m. morsitans* flies and a weekly plasma collection over a period of 6 weeks.

### Serological analyses

IgG responses in the exposed animals were analyzed by indirect ELISA against rTsal1 and saliva from different hematophagous insects. For this purpose, polystyrene 96 well plates (Thermo Scientific NUNC MaxiSorp Surface) were coated overnight at 4°C with 200 ng antigen (*G. morsitans*, *S. calcitrans* or *T. yao* saliva or rTsal1) per well in 0.1 M NaHCO_3_ (pH 8.3). Plates were overcoated for 1 h with 10% fetal bovine serum (FBS) at ambient temperature. Serial half plasma dilutions starting from 1∶100 in assay diluent (PBS/10%FBS) were applied for 2 h to antigen and FBS-coated wells. For the analysis of porcine plasma samples, 20% Top10F' *E. coli* soluble extract was added to the assay diluent to reduce unspecific binding to the antigenic coat. Based on the plasma dilution experiments, a 1∶1600 dilution was chosen for the time course analyses. Specific immunoglobulin detection was achieved using horseradish peroxidase conjugated detection antibodies. For the detection of mouse and porcine IgGs respectively a 1∶1000 diluted rabbit F(ab)_2_ anti-mouse IgG (STAR13B, Serotec) and a rabbit anti-pig IgG conjugate (A5670, Sigma) 1∶4000 diluted in PBS/10%FBS were used. Detection was with TMB substrate (3,3′,5,5′-Tetramethylbenzidine, Sigma) and reactions were stopped by the 1∶3 addition of 1N H_2_SO_4_. Optical density (O.D.) was measured using a Multiskan Ascent plate reader (Thermo) at a 450 nm wavelength.

### Data analysis

Antigen-specific responses were expressed as the ΔO.D. between antigen and non-antigen-coated wells. Statistical analyses were performed in SAS Version 9.3 (SAS Institute Inc., Cary, NC, USA). The effect of tsetse fly exposure intensity and boosting on anti-*G. m. morsitans* saliva and anti-rTsal1 immune responses in mice and pigs was analysed over the entire sampling period based on a mixed model with animal as random effect and challenge, time and their interaction as categorical fixed effects and F-tests were performed at the 5% significance level. Pairwise comparisons were performed using Tukey's multiple comparisons technique to adjust the significance level. Cross-reactivity of immune responses induced by different *Glossina* species and other hematophagous insects was analysed using a linear fixed effects model using the 1∶100 or 1∶1600 dilution with challenge as categorical fixed effect. Pairwise comparisons were performed with control using Dunnett's multiple comparisons technique to adjust the significance level. Intra-laboratory repeatability of the ELISA tests was assessed by the non-parametric Spearman correlation test. Sensitivity and specificity of the assays were assessed by receiver operating characteristic (ROC) curve analysis of the ΔO.D. values of exposed and non-exposed animals, starting from 3 weeks after the initial exposure. The area under the ROC curve (AUC) was used as a global index of diagnostic accuracy. Kinetics of the IgG clearance in mice was assessed in five mice from the multiple exposure schemes with sufficient remaining plasma for the different timepoints and using the plasma sample with the highest antibody titer (set as 100%) to prepare a standard curve. Percent decrease in antibody titers over time was assessed by a two-phase non-linear regression allowing the estimation of the antibody half-life.

## Results

### Serological responses in mice exposed to *Glossina morsitans*

**Figure 1:**
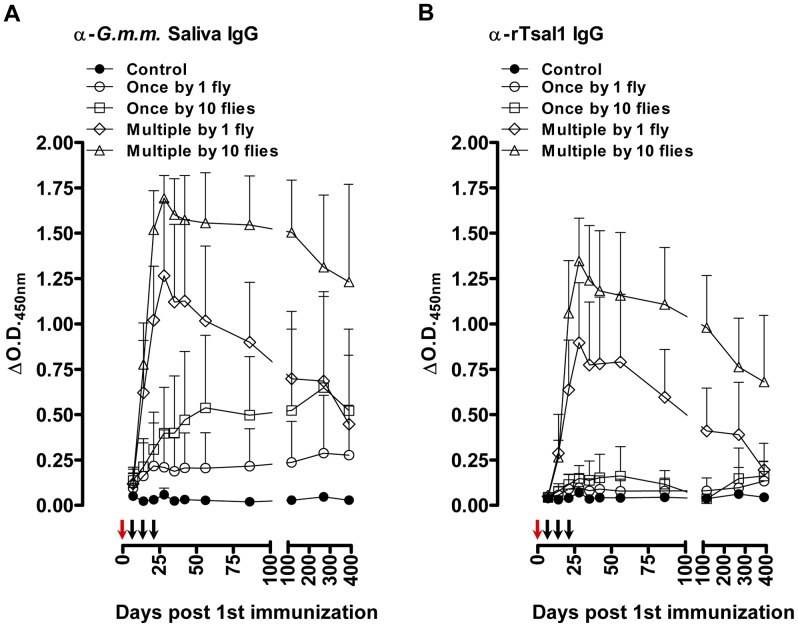
Tsetse fly induced humoral responses in mice. Long-term follow-up of (**A**) the anti-*G. m. morsitans* saliva IgG responses and (**B**) the anti-rTsal1 IgG responses in mice (*n* = 8/group) exposed to 4 different tsetse fly biting intensities (1 versus 10 flies) and frequencies (single versus weekly exposure for 3 weeks). Arrows indicate the tsetse exposure regimen for single exposed (red arrows) and multiple exposed mice (red and black arrows). Presented data are the mean ΔO.D._450 nm_ values and the 95% CI obtained with a 1∶1600 dilution of the individual plasmas.

**Figure 2:**
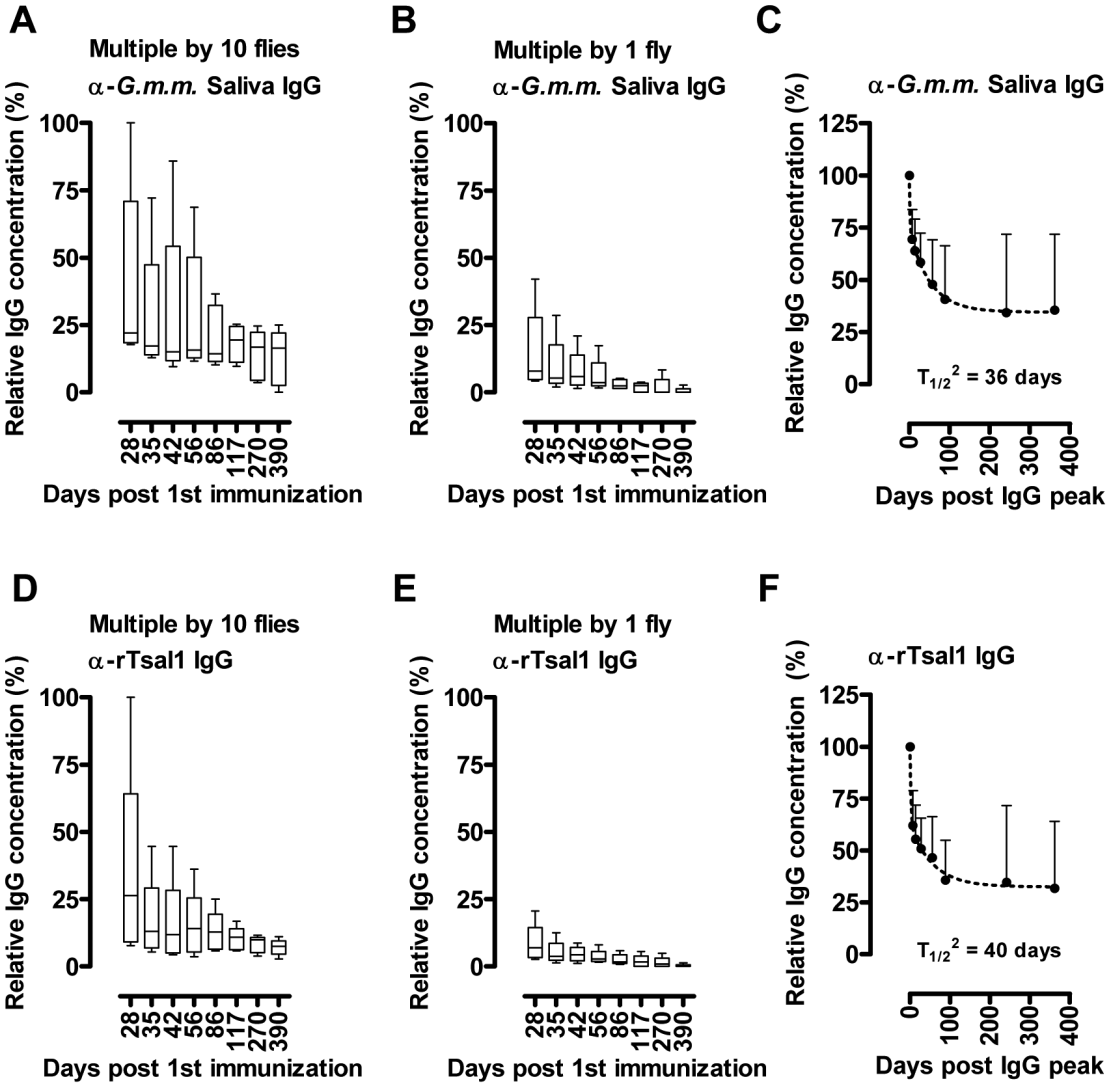
Antibody kinetics in tsetse exposed mice. Evolution over time in the absence of tsetse fly challenge (from day 28 onwards) of the anti-*G. m. morsitans* saliva IgG concentrations (**A, B, C**) and the anti-rTsal1 IgG concentrations (**D, E, F**) in mice (*n* = 5/group) exposed multiple times to a single fly (**B, E**) or 10 flies (**A, D**). Presented data are the IgG concentrations relative to the highest responder plasma and expressed as percent based on a standard dilution series. Box plots represent the means, with minimum and maximum and the 75-25% percentiles. The average antibody half-life (T_1/2_^2^, **C, F**) over the 2 exposure groups (*n* = 10) was determined by a two-phase non-linear decay regression (dotted line) of the declining antibody concentrations relative to the concentration recorded after cessation of the tsetse exposure (day 28 set as starting concentration of 100%). Scatter plots indicate the mean concentrations with the 95% CI.

**Figure 3:**
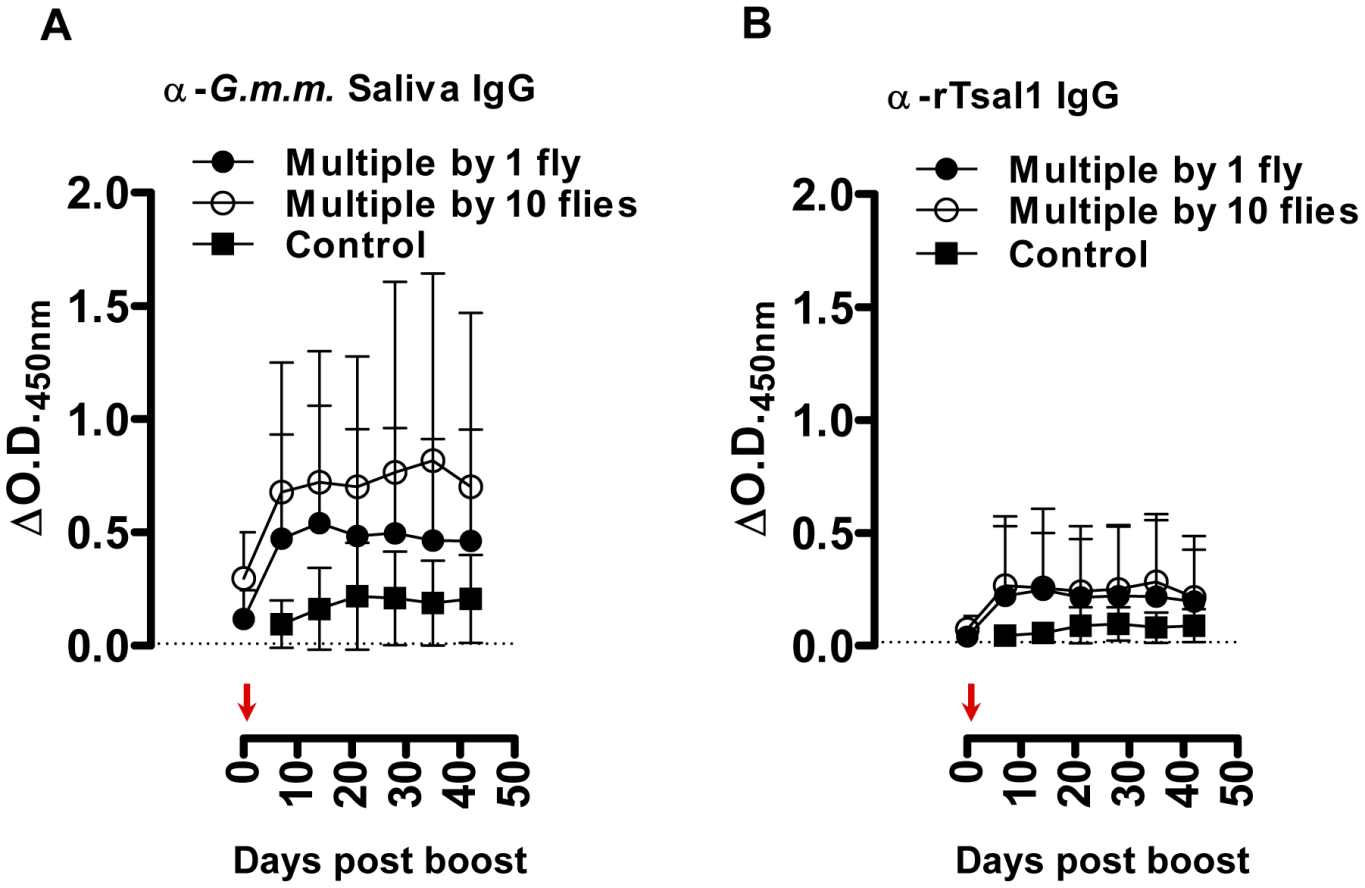
Effect of boosting in mice after a long period of non-exposure. Evolution of the anti-*G. m. morsitans* saliva IgG responses (**A**) and the anti-rTsal1 IgG repsonses (**B)** was evaluated in mice (*n* = 6/group) originally exposed to the bites of a single or 10 flies and re-exposed to a single tsetse fly bite after a 1-year period of non-exposure (indicated by an arrow). Controls are the naive mice exposed to a single bite as depicted in Figure 1. The original control animals (*n* = 3) were not exposed and served as negative controls (dotted line). Presented data are mean ΔO.D._450 nm_ values with the 95% CI obtained with a 1∶1600 dilution of the individual plasmas.

Induction of specific antibody responses was assessed by indirect ELISA in mice following different regimens of exposure to *Glossina morsitans morsitans* bites. Anti-*G. m. morsitans* saliva and anti-rTsal1 IgGs were detectable within 7 days after the initial exposure and remained persistently detectable up to 390 days (Figure 1). The detected IgG titers against both antigens correlated with the different intensities of tsetse fly exposure. Differences in number of flies (1 versus 10 flies) as well as differences in frequency (single versus weekly exposure over 3 weeks) were detected by ELISA. Exposure to a single tsetse fly bite was sufficient to induce slightly elevated levels of antibodies against *G. m. morsitans* saliva and rTsal1 (Figure 1), although the recorded differences with control plasmas were not significant (*p* = 0.9843 and *p* = 0.6146 for the anti-rTsal1 and anti-*G. m. morsitans* saliva tests respectively). Single exposure of mice to 10 flies resulted in significantly increased reactivity against *G. m. morsitans* saliva (*p* = 0.0160) but not against rTsal1 (*p* = 0.8618). With both antigens, statistically significant differences were recorded considering the entire sampling period between control mice and mice subjected to the repeated exposure to 1 and 10 flies (*p*<0.0001). Both the anti-rTsal1 and the anti-*G. m. morsitans* saliva ELISA test were able to differentiate between these two repeated tsetse exposure schemes (*p*<0.0001 and *p* = 0.0012 respectively). Based on a standard curve generated using the plasma sample with the highest IgG titer (i.e. day 28, mouse exposed to the most intense biting regimen), antibody half-lives in five mice exposed multiple times to a single fly or 10 flies were determined by two-phase decay regressions (Figure 2). The average half-lives (T_1/2_^2^) of the anti-saliva and anti-rTsal IgGs in a second phase of decay after a first phase immediately after cessation of tsetse exposure were respectively 36 and 40 days (Figure 2). There was a significant individual variation within the different exposure groups with up to a 10-fold difference between the strongest and weakest responder. On average, a 3 to 4-fold difference in peak IgG titer was recorded between mice exposed to a low (multiple bites by a single fly) and high exposure regimen (multiple bites by 10 flies). The antibodies appeared relatively persistent over an evaluation period of more than 1 year. Following a long period of non-exposure, the bite of a single tsetse fly was sufficient to boost the anti-saliva and anti-rTsal1 IgG titers. This boosting appeared independent of the previous exposure intensity (Figure 3) as statistical analysis over the 6-week sampling period revealed the inability of the saliva and rTsal1-based tests to differentiate mice subjected to a low or high initial exposure regimen (*p* = 0.4141 and *p* = 0.9609 respectively). Antibody titers against both antigens reached a plateau within 7 days after re-challenge, while in naive animals the responses were slightly lower (*p* = 0.0833 and *p* = 0.1925 for the anti-saliva and anti-rTsal1 IgG levels respectively) and only reached peak titers within 4 weeks after initial exposure (Figure 3).

A strong correlation between anti-saliva and anti-rTsal1 ELISA results was recorded with a Spearman correlation coefficient *r* of 0.92. Intra-laboratory repeatability of the anti-saliva and anti-rTsal1 detection assays was excellent (Spearman *r* = 0.98). A comparison of the diagnostic value of the anti-saliva and rTsal1 indirect ELISA was conducted using ROC curve analysis. Comparison of the area under the curve (AUC), based on the mouse plasmas from the experiment presented in Figure 1 and collected at least 3 weeks after the initial tsetse fly exposure, indicated that the anti-tsetse saliva ELISA has a better test performance than the rTsal1-based assay (AUC 0.94 versus 0.82, Figure S1).

### Serological responses of mice exposed to various *Glossina* species

**Figure 4:**
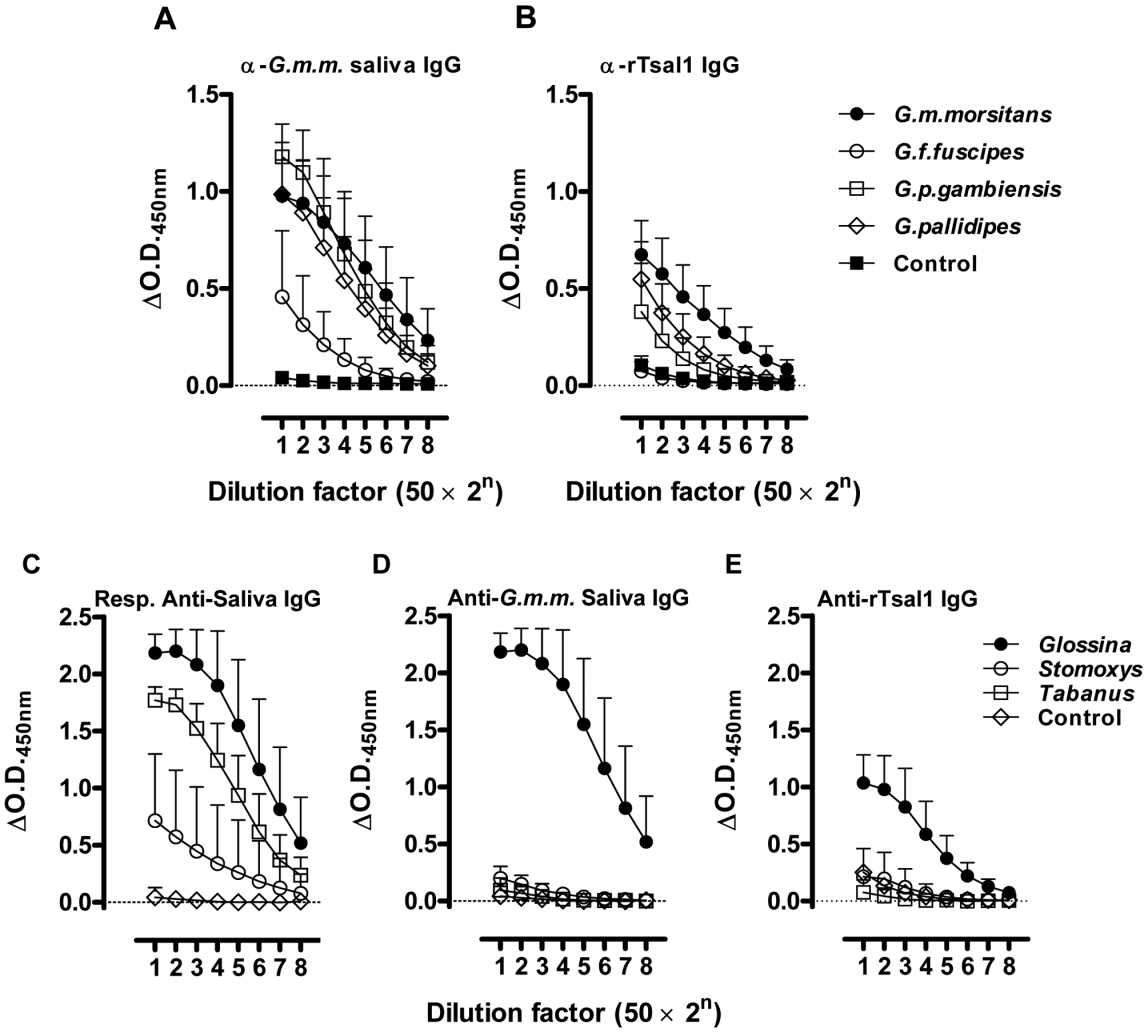
Detection of humoral responses induced by various hematophagous insects. Serially diluted plasmas of control mice and mice repeatedly exposed to the bites of *G. morsitans morsitans*, *G. fuscipes fuscipes*, *G. palpalis gambiensis* and *G. pallidipes* (*n* = 5/group) were tested in the *G. m. morsitans* saliva-based (**A**) and rTsal1-based IgG detection ELISA (**B**). Serially diluted plasmas of control mice and mice experimentally immunized with harvested *G. m. morsitans*, *Stomoxys calcitrans* and *Tabanus yao* saliva (*n* = 6/group) were analysed for responses against the respective saliva extracts (**C**) and were tested in the *G. m. morsitans* saliva-based (**D**) and rTsal1-based IgG detection ELISA (**E**). Presented data are the mean ΔO.D._450 nm_ values with the 95% CI.

Mice were repeatedly exposed to the bites of various *Glossina* species (*G. m. morsitans*, *G. p. gambiensis*, *G. pallidipes* and *G. f. fuscipes*). It was noted that the feeding performance of *G. fuscipes* on mice was inferior to those of the other species, resulting in a high mortality of these flies when maintained on mice. *G. m. morsitans* saliva and rTsal1 were next used as antigens in indirect ELISA to detect the elicited antibodies. A marked cross-reactivity of the anti-saliva IgGs elicited by three *Glossina* species (*G. m. morsitans*, *G. p. gambiensis* and *G. pallidipes*, *p*<0.0001 at the 1∶100 plasma dilution) and a relatively weaker cross-reactivity of *G. f. fuscipes* (*p* = 0.0053) was detected with *G. m. morsitans* saliva as antigen (Figure 4A&B). Similarly, antibodies induced by exposure to *G. m. morsitans*, *G. p. gambiensis* and *G. pallidipes* significantly reacted with rTsal1 (respectively *p*<0.0001, *p* = 0.0157 and *p* = 0.0002), but no cross-reaction with rTsal1 was detected in mice exposed to *G. f. fuscipes* (*p* = 0.9883). This indicated that a number of antigens are sufficiently conserved in the saliva of several *Glossina* species to allow multi-species detection of exposure using *G. m. morsitans* saliva. The rTsal1 also enabled detection of exposure of mice to various tested tsetse fly species, except for *G. fuscipes* (Palpalis subgenus).

### Serological responses of mice exposed to various hematophagous insect species

Mice were experimentally immunized by 4 intradermal injections with the saliva of stable flies (*Stomoxys calcitrans*) or horse flies (*Tabanus yao*) and compared with control mice and mice that were immunized with tsetse fly saliva following the same immunization protocol. Immunization with *Tabanus* saliva did not result in IgGs that reacted with rTsal1 and total *G. morsitans* saliva as coating antigens (*p* = 0.7882 and *p* = 0.7637 at the 1∶100 plasma dilution, Figure 4D&E). However, a slight increase that did not reach statistical significance was observed in responsiveness of the *Stomoxys* exposed plasmas in the rTsal1 and saliva-based assays (*p* = 0.0837 and *p* = 0.0639 respectively). At a standard 1∶1600 dilution, no cross-reaction of the *Stomoxys* exposed plasmas with rTsal1 and saliva was detected (*p* = 0.9920 and *p* = 0.9915 respectively). Analysis of the published salivary transcriptome of *Stomoxys calcitrans* suggested the presence of a Tsal1 homologue from which a truncated sequence was published (GenBank Accession N°: ACN69159, [26]). Within this region, only 36% identity in amino acid sequence was found, which could explain the slightly elevated anti-rTsal1 reactivity of *Stomoxys* exposed mice.

### Serological responses in tsetse fly exposed pigs

**Figure 5:**
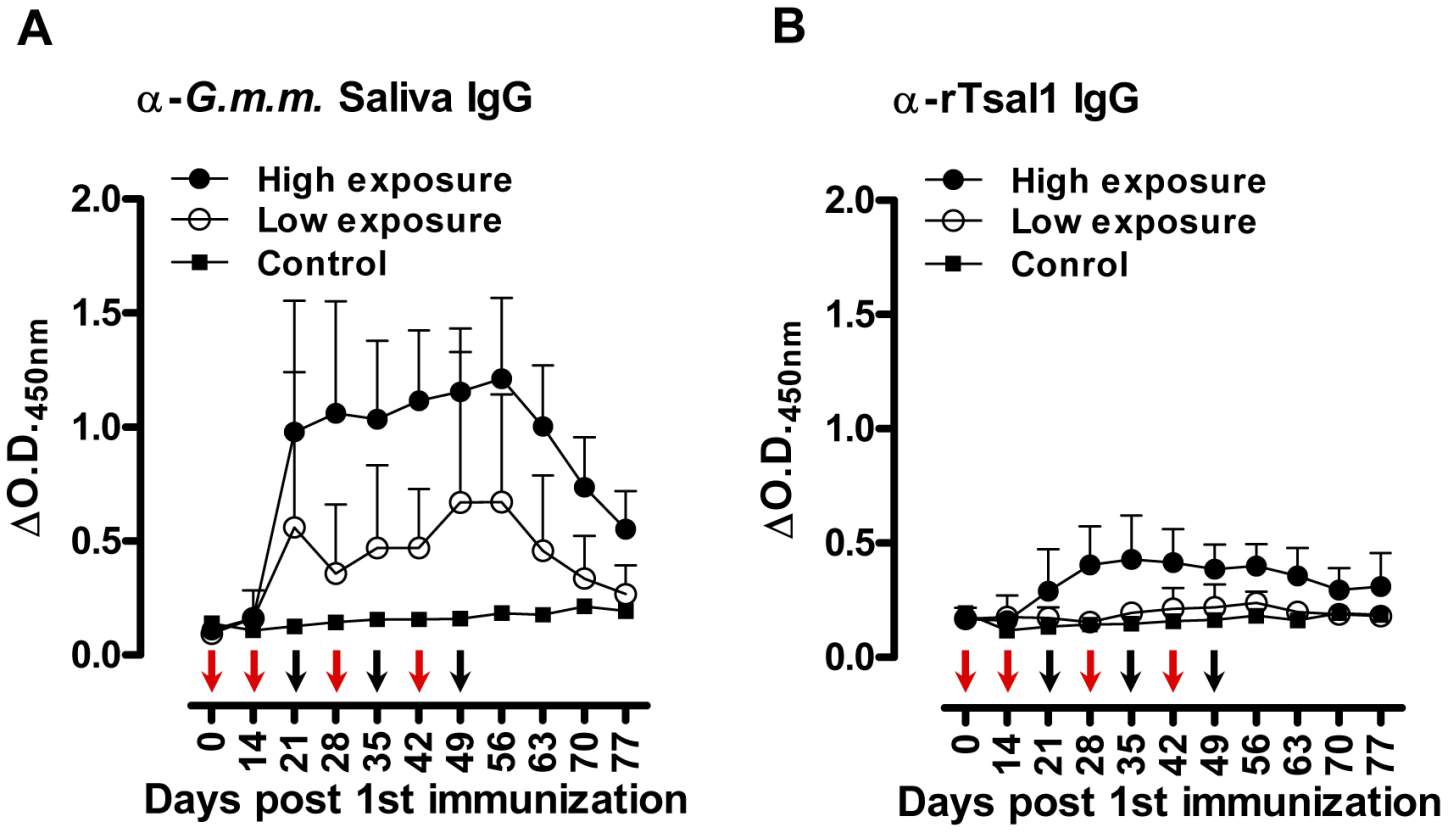
Tsetse fly induced humoral responses in pigs. Follow-up of the anti-*G. m. morsitans* saliva IgG responses (**A**) and the anti-rTsal1 IgG responses (**B**) in pigs exposed to 2 different tsetse fly biting intensities (two-weekly exposure for 6 weeks to 3 flies (low exposure group, n = 4) or weekly exposure for 7 weeks to 30 flies (high exposure group, n = 5)). One non-exposed animal was included as a negative control. Arrows indicate the tsetse exposure regimen for the low exposure (red arrows) and high exposure group (red and black arrows). Presented data are the mean ΔO.D._450 nm_ values and the 95% CI obtained with a 1∶1600 dilution of the individual plasmas.

**Figure 6:**
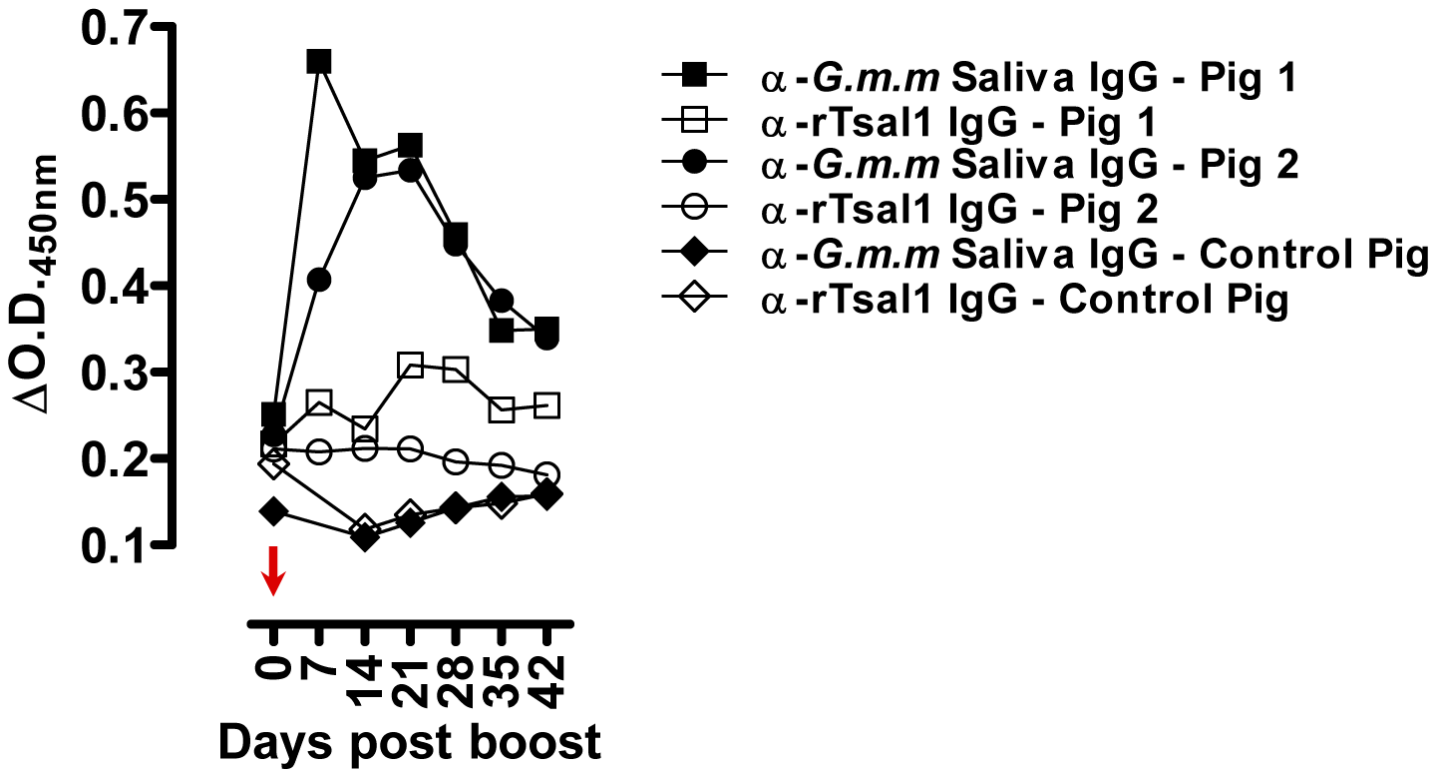
Effect of boosting in pigs after a period of non-exposure. Evolution of the anti-*G. m. morsitans* saliva IgG responses and the anti-rTsal1 IgG repsonses was evaluated in pigs (*n* = 2) originally exposed two-weekly for 6 weeks to the bites of 3 flies ( = low exposure group) and re-exposed to the bites of 10 flies (indicated by an arrow) after a 2-month period of non-exposure. One non-exposed animal sampled during the priming experiment (Figure 5) served as a negative control. Presented data are mean ΔO.D._450 nm_ values with the 95% CI obtained with a 1∶1600 dilution of the individual plasmas.

Pigs were experimentally exposed to a low or a high tsetse fly bite regimen, followed by assessment of the antibody production. As compared to the mouse indirect ELISA, we have modified the porcine assay by including *E. coli* soluble extract in the sample diluent as described elsewhere [25] to reduce the background that was observed particularly onto the rTsal1 antigen which was produced in a bacterial expression system. Under the used assay conditions, anti-rTsal1 and anti-saliva IgGs were detected from day 21 after the first tsetse exposure onwards (Figure 5). The specific IgG titers in pigs correlated with the intensity of tsetse exposure. Anti-rTsal1 and anti-tsetse saliva IgGs were elevated in tsetse exposed groups as compared to the non-exposed control pig and pre-immune plasmas, with significant differences for the high exposure group (*p* = 0.0216 and *p* = 0.0030 respectively). The repeated exposure of pigs to 30 flies resulted in higher anti-rTsal1 and anti-tsetse saliva IgG titers as compared to the pigs exposed to a low tsetse challenge by 3 flies (*p* = 0.0014 and *p* = 0.0024 respectively). However, exposure to the low exposure scheme did not result in significantly elevated responses in both the rTsal1 and saliva-based ELISA (*p* = 0.8844 and *p* = 0.4261 respectively, Figure 5). Boosting of 2 pigs from the low exposure group after a 2-month non-exposure period by the bites of 10 flies resulted in elevated anti-saliva IgG titers but was only weakly detectable using the rTsal1-based ELISA and with higher individual variation (Figure 6). In general, a good correlation between anti-saliva and anti-rTsal1 ELISA results, obtained for the samples from the tsetse fly exposure experiment presented in Figure 5, was recorded with a Spearman correlation coefficient *r* of 0.79. Intra-laboratory repeatability of the anti-saliva and anti-rTsal1 detection assays was adequate (Spearman *r* = 0.94). Comparison of the area under the curve (AUC) for the anti-tsetse saliva and anti-rTsal1 ELISA ROC curve indicated that the assay based on total *G. morsitans* saliva has a better average performance than the anti-rTsal1 IgG detection test (AUC 0.96 versus 0.83, Figure S2).

## Discussion

Assessment of exposure of populations at risk to the bites of tsetse fly vectors could be an important step towards improved control of both HAT and AAT. Given that novel innovative vector control strategies are being developed (e.g. miniaturized insecticide-treated targets [27], the release of sterile male insects [8], [9]) and deployed on increasingly large scales on the African continent, monitoring the impact of these new as well as conventional interventions on actual exposure to tsetse fly bites is a logical follow-up. The tsetse fly species implicated in the transmission of trypanosomes are not uniform throughout the African continent as more than 30 tsetse species and subspecies exist that play differential roles in parasite transmission, have preferences for specific biotopes and display specific host feeding preferences. A number of species are strongly implicated in HAT transmission, such as flies of the Morsitans subgenus (e.g. used in this study: *G. morsitans morsitans*, *G. pallidipes*) and flies of the Palpalis group (e.g. used in this study: *G. palpalis gambiensis*, *G. fuscipes fuscipes*). In case of AAT, a large panel of tsetse species and other sympatric hematophagous arthropods are respectively involved in biological and mechanical transmission. Especially stable flies (*Stomoxys* sp.) and horse flies (*Tabanus* sp.) play a role in mechanical transmission of animal trypanosomes such as *T.vivax*. However, these insects are not able to biologically transmit trypanosomes as the parasite cannot complete its life cycle in these insects.

Research on a number of hematophagous arthropod vectors has resulted in the concept of exploiting salivary components as specific biomarkers of exposure [15], [28], [29], [30], [31], [32], [33]. An advantage of this approach is that relatively simple serological tests could provide information on actual exposure to the bites of disease vectors and provide a risk indicator of contracting a vector-transmitted disease without the need of strenuous entomological surveys. Whole salivary gland extracts of sand flies [33], [34], [35], triatomine bugs [30], [31] and various mosquito species [36], [37], [38] can be used to assess biting exposure. It has been suggested that this type of serological approach would enable the detection of very low levels of exposure that could remain undetected by entomological trappings [39]. Also differences in exposure level due to vector control interventions (e.g. insecticide treated bednets) could be elucidated on the basis of salivary proteins as immunological probes [29], [34], [40], [41]. While total salivary extracts can be used in various ELISA and immunoblot formats, some studies have moved towards the use of recombinant proteins or peptides which could lead to the development of more standardized immune assays [29], [42], [43], [44], [45], [46], [47], [48], [49]. Several strategies could be envisaged either aiming at species-specific or pan-species exposure detection.

Studies using various tsetse fly species have shown that salivary components are immunogenic in mice, rabbits, cattle and humans [15], [17], [18], [19], [20], [23]. Immunoblotting of salivary proteins separated on 1D or 2D protein gels have highlighted immunogenic proteins corresponding to several protein families including endonuclease (Tsal), adenosine deaminase (TSGF), 5′nucleotidase (5′Nuc) and Antigen 5 (Ag5) related proteins [19]. In addition, the immunogenic nature of *G. m. morsitans sgp1*, *sgp2* and *sgp3* resulted in their identification on the basis of immune screening of a phage cDNA expression library [50]. We have previously observed by Western blot analysis that human plasma samples collected in Tororo (Uganda, [51]) where *G.fuscipes fuscipes* is the predominant tsetse fly, commonly recognized the 43–45 kDa Tsal proteins in *G. m. morsitans* saliva [15]. This suggested that *G. m. morsitans* Tsal-based immune screening of these Ugandan samples cross-detected exposure to *G. fuscipes fuscipes*, responsible for *Trypanosoma brucei rhodesiense* transmission in that area [52]. Plasma samples from tsetse fly exposed individuals in Guinean HAT foci also displayed strong reactivity against the highly abundant Tsal proteins in *G. palpalis gambiensis* saliva in immunoblots [19]. As the Tsal protein band is commonly recognized in samples from *Glossina*-exposed humans [15], [19], Tsal proteins could have the potential of a pan-*Glossina* species exposure marker. However, reactivity with Tsal1 and Tsal2 was also observed with a pool of human control plasmas from Bobo-Dioulasso (Burkina Faso) [19]. Based on our current study we anticipate that the inclusion criteria for these urban unexposed plasma donors (not having traveled outside of the city for at least three months, [21]) might have been insufficiently stringent to exclude circulating anti-Tsal antibodies. Indeed, tsetse flies are present at sites outside the city and given the high immunogenicity and anticipated long antibody persistence, this could explain the documented positive reactions in the immunoblots.

Production of a recombinant version of the Tsal1 protein allowed evaluating its effectiveness for detecting tsetse fly exposure in an antibody-detection ELISA. By experimentally exposing mice and pigs to different tsetse fly biting regimens, the high immunogenicity of total *G. m. morsitans* saliva and Tsal1 as a major constituent was confirmed. In mice, a single bite was proven sufficient to induce a detectable immune response in naive animals and to boost antibody levels in previously exposed animals. Kinetics of the antibody clearance was assessed in mice over a >1 year period, which revealed a long persistence with an average half-life of 36–40 days. In pigs, the apparent antibody clearance rate was faster and although the time of sampling and the number of experimental animals was limited, the anti-saliva IgG half-life could be estimated to be 15 days (data not shown). This clearance rate was consistent with the fast decline of anti-*G. m. submorsitans* saliva IgG levels observed in cattle within 10 weeks after cessation of tsetse exposure [20]. Both in mice and pigs, boosting of prior induced anti-saliva immunity was observed within 7 days by the anti-*G. m. morsitans* saliva and anti-rTsal1 ELISA. However, with the used sample sizes and experimental conditions, both the rTsal1 and saliva-based ELISA were unable to statistically differentiate the control pig from pigs exposed to a low tsetse fly challenge. Interestingly, the boosting of immunity in mice seemed independent of the previous exposure intensity. As such, surveys based on rTsal1 or total *G. m. morsitans* saliva in tsetse fly control intervention zones would give information on the actual tsetse exposure intensity rather than history and could provide a tool to monitor re-invasion. Consistent with this, serological results for cattle in South-West Burkina Faso based on *G. palpalis gambiensis* whole saliva extract related to the seasonality which directly impacts the intensity of host/vector contact [20]. Also humans living in HAT endemic areas display significant differences in anti-*G. p. gambiensis* saliva IgGs depending on the study site [19]. Similar results were obtained with a peptide derived from the adenosine deaminase-related protein TSGF1, although obtained ELISA signals seemed very low [21]. In both the cattle and human ELISA tests, large inter-individual differences were observed [15], [19], [20], [21]. It should be noted that in our study the individual variation in antibody titers in the different exposure groups also varied significantly. In controlled experimental conditions with outbred mice, we recorded up to 10-fold differences in anti-saliva and anti-rTsal1 antibody concentrations between the strongest and weakest responders. Consequently, using the tsetse salivary antigens as immunological probes has the limitation of inherent variability due to individual differences in immunological responsiveness. Nevertheless, we observed a clear overall difference in anti-rTsal1 and anti-saliva IgG levels between the different exposure groups of mice and pigs, with an average 3–4 fold difference in specific antibody concentration between a low exposure (repeated exposure to 1 fly for mice or to 3 flies for pigs) and a high exposure regimen (repeated exposure to 10 flies for mice or to 30 flies for pigs). This is in line with observations made for cattle that were experimentally exposed to different biting intensities [20]. Beside population-level studies, individual serological follow-up of selected animals or sentinel animals using the recombinant Tsal1 or the total *G. morsitans* saliva could provide a measure for tsetse prevalence, provide evidence for the impact of an intervention strategy or could be a sensitive tool to detect re-invasion of previously cleared areas or the efficacy of a barrier protecting a cleared area. Here, rTsal1 and total *G. morsitans* saliva seem to provide an indication of exposure to a broad range of *Glossina* species. Only for *G. f. fuscipes*, detection of induced IgGs is hampered which could relate to the genetic distance of this species as member of the Palpalis group (although *G. palpalis gambiensis* was efficiently cross-detected). Nevertheless, serological responses against rTsal1 were detected in humans from Uganda principally exposed to *G. fuscipes*, which suggests that sensitivity and specificity parameters related to a single antigen may vary considerably depending on the host species. It also remains to be evaluated whether the intensity and persistence of the anti-Tsal1 IgG responses would not result in a saturation in populations persistently exposed to tsetse bites. This could possibly limit application of the rTsal1-based assay to examining subjects in tsetse-free areas that are at risk of invasion or to monitoring sentinel animals in order to evaluate tsetse presence in endemic regions.

Exposure to the saliva of other hematophagous insects (*Tabanus* and *Stomoxys* sp.) that may be abundant in areas where tsetse flies are present, revealed that *G. m. morsitans* saliva did not yield false positive signals. Observations of *Stomoxys* and *Tabanus* exposed cattle revealed that also *G. m. submorsitans* saliva shares this feature of specificity, while *Glossina tachinoides* and *G. palpalis gambiensis* saliva yielded unspecific reactions with *Tabanus* exposed plasmas [20]. When using rTsal1 as an antigen, a weak unspecific signal was observed with the 1∶100 to 1∶800 diluted plasmas of mice exposed to *Stomoxys calcitrans* saliva which could be due to the presence of a homologue (GenBank Accession N°: ACN69159, [26]) with relatively limited degree of identity to Tsal1. Given the strong immunogenicity of the Tsal proteins and based on our ELISA analyses with the experimentally exposed pigs and mice we propose to use a 1∶1600 plasma dilution in favor of an increased specificity and an overall reduced risk of detecting cross-reactive antibodies. For the porcine ELISA, *E. coli* soluble extract was added to the sample diluent to increase the specificity similar to what was proposed for other pig serological tests [25]. Under these stringent experimental conditions, the recombinant Tsal1 was able to detect the high levels of exposure to tsetse fly bites and with a good correlation with the data obtained for total *G. m. morsitans* saliva as an antigen.

Collectively and combined with our previous analyses using a large panel of East-African human plasmas [15], this study indicates that a recombinant version of the *G. m. morsitans* Tsal1 fulfills the criteria of a candidate exposure biomarker for a broad range of tsetse fly species. We believe that the high sensitivity of the rTsal antigen and the broad species recognition could be an added value to the immunoassays that are tested in the framework of other studies based on tsetse salivary peptides.

## Supporting Information

Figure S1 **Repeatability and specificity/sensitivity analysis of the antibody detection test in mice.** Scatter plot analysis of the anti-*G. m. morsitans* saliva IgG responses (**A**) and the anti-rTsal1 IgG repsonses (**B**) (ΔO.D._450 nm_) in two separate tests performed on a panel of 1∶1600 diluted mouse plasma samples (*n* = 352). Test repeatability was analyzed by the non-parametric Spearman correlation test. Sensitivity and specificity of the two assays were assessed by receiver operating characteristic (ROC) curve analysis of the ΔO.D. values of exposed and non-exposed mice (**C**). The area under the ROC curve (AUC) is reported as a measure for the test performance.
(TIF)

Figure S2 **Repeatability and specificity/sensitivity analysis of the antibody detection test in pigs.** Scatter plot analysis of the anti-*G. m. morsitans* saliva IgG responses (**A**) and the anti-rTsal1 IgG repsonses (**B**) (ΔO.D._450 nm_) in two separate tests performed on a panel of 1∶1600 diluted porcine plasma samples (*n* = 80). Test repeatability was analyzed by the non-parametric Spearman correlation test. Sensitivity and specificity of the two assays were assessed by receiver operating characteristic (ROC) curve analysis of the ΔO.D. values of exposed and non-exposed mice (**C**). The area under the ROC curve (AUC) is reported as a measure for the test performance.
(TIF)

## References

[1] Simarro PP, Cecchi G, Paone M, Franco JR, Diarra A, et al (2010) The Atlas of human African trypanosomiasis: a contribution to global mapping of neglected tropical diseases. Int J Health Geogr 9: 57. 21040555 10.1186/1476-072X-9-57 PMC2988709

[2] Aksoy S (2011) Sleeping sickness elimination in sight: time to celebrate and reflect, but not relax. PLoS Negl Trop Dis 5: e1008. 21364971 10.1371/journal.pntd.0001008 PMC3042998

[3] Simarro PP, Jannin J, Cattand P (2008) Eliminating human African trypanosomiasis: where do we stand and what comes next? PLoS Med 5: e55. 18303943 10.1371/journal.pmed.0050055 PMC2253612

[4] Cattand P (2010) Food and Agriculture Organization, Programme Against African Trypanosomiasis (Paat) (2010) Linking sustainable human and animal African trypanosomiasis control with rural development strategies. PAAT technical and scientific series 10. Food and Agriculture Organization of the United Nations 76.

[5] Vreysen MJ, Seck MT, Sall B, Bouyer J (2013) Tsetse flies: their biology and control using area-wide integrated pest management approaches. J Invertebr Pathol 112 Suppl: S15–25. 22878217 10.1016/j.jip.2012.07.026

[6] Schofield CJ, Kabayo JP (2008) Trypanosomiasis vector control in Africa and Latin America. Parasit Vectors 1: 24. 18673535 10.1186/1756-3305-1-24 PMC2526077

[7] Torr SJ, Hargrove JW, Vale GA (2005) Towards a rational policy for dealing with tsetse. Trends Parasitol 21: 537–541. 16140579 10.1016/j.pt.2005.08.021

[8] Vreysen MJ, Saleh KM, Ali MY, Abdulla AM, Zhu ZR, et al (2000) *Glossina austeni* (Diptera: Glossinidae) eradicated on the island of Unguja, Zanzibar, using the sterile insect technique. J Econ Entomol 93: 123–135. 14658522 10.1603/0022-0493-93.1.123

[9] Sow A, Sidibe I, Bengaly Z, Bance AZ, Sawadogo GJ, et al (2012) Irradiated male tsetse from a 40-year-old colony are still competitive in a Riparian forest in Burkina Faso. PLoS One 7: e37124. 22590652 10.1371/journal.pone.0037124 PMC3349643

[10] Fontaine A, Diouf I, Bakkali N, Misse D, Pages F, et al (2011) Implication of haematophagous arthropod salivary proteins in host-vector interactions. Parasit Vectors 4: 187. 21951834 10.1186/1756-3305-4-187 PMC3197560

[11] Alves-Silva J, Ribeiro JM, Van Den Abbeele J, Attardo G, Hao Z, et al (2010) An insight into the sialome of *Glossina morsitans morsitans* . BMC Genomics 11: 213. 20353571 10.1186/1471-2164-11-213 PMC2853526

[12] Caljon G, De Ridder K, De Baetselier P, Coosemans M, Van Den Abbeele J (2010) Identification of a tsetse fly salivary protein with dual inhibitory action on human platelet aggregation. PLoS One 5: e9671. 20351782 10.1371/journal.pone.0009671 PMC2843633

[13] Caljon G, Van Den Abbeele J, Stijlemans B, Coosemans M, De Baetselier P, et al (2006) Tsetse fly saliva accelerates the onset of *Trypanosoma brucei* infection in a mouse model associated with a reduced host inflammatory response. Infect Immun 74: 6324–6330. 16954393 10.1128/IAI.01046-06 PMC1695494

[14] Cappello M, Bergum PW, Vlasuk GP, Furmidge BA, Pritchard DI, et al (1996) Isolation and characterization of the tsetse thrombin inhibitor: a potent antithrombotic peptide from the saliva of *Glossina morsitans morsitans* . Am J Trop Med Hyg 54: 475–480. 8644901 10.4269/ajtmh.1996.54.475

[15] Caljon G, Van Den Abbeele J, Sternberg JM, Coosemans M, De Baetselier P, et al (2006) Tsetse fly saliva biases the immune response to Th2 and induces anti-vector antibodies that are a useful tool for exposure assessment. Int J Parasitol 36: 1025–1035. 16777113 10.1016/j.ijpara.2006.05.002

[16] Caljon G, De Ridder K, Stijlemans B, Coosemans M, Magez S, et al (2012) Tsetse salivary gland proteins 1 and 2 are high affinity nucleic acid binding proteins with residual nuclease activity. PLoS One 7: e47233. 23110062 10.1371/journal.pone.0047233 PMC3479092

[17] Poinsignon A, Remoue F, Rossignol M, Cornelie S, Courtin D, et al (2008) Human IgG antibody response to *Glossina* saliva: an epidemiologic marker of exposure to Glossina bites. Am J Trop Med Hyg 78: 750–753. 18458309

[18] Poinsignon A, Cornelie S, Remoue F, Grebaut P, Courtin D, et al (2007) Human/vector relationships during human African trypanosomiasis: initial screening of immunogenic salivary proteins of *Glossina* species. Am J Trop Med Hyg 76: 327–333. 17297044

[19] Dama E, Cornelie S, Bienvenu Somda M, Camara M, Kambire R, et al (2013) Identification of *Glossina palpalis gambiensis* specific salivary antigens: towards the development of a serologic biomarker of human exposure to tsetse flies in West Africa. Microbes Infect 15: 416–427. 23500186 10.1016/j.micinf.2013.03.001

[20] Somda MB, Bengaly Z, Dama E, Poinsignon A, Dayo GK, et al (2013) First insights into the cattle serological response to tsetse salivary antigens: A promising direct biomarker of exposure to tsetse bites. Vet Parasitol 197: 332–340. 23800781 10.1016/j.vetpar.2013.05.018

[21] Dama E, Cornelie S, Camara M, Somda MB, Poinsignon A, et al (2013) Identification of a Candidate Synthetic Peptide (Tsgf1) to Monitor Human Exposure to Tsetse Flies in West Africa. PLoS Negl Trop Dis 7: e2455. 24086785 10.1371/journal.pntd.0002455 PMC3784472

[22] Caljon G, Broos K, De Goeyse I, De Ridder K, Sternberg JM, et al (2009) Identification of a functional Antigen5-related allergen in the saliva of a blood feeding insect, the tsetse fly. Insect Biochem Mol Biol 39: 332–341. 19507303 10.1016/j.ibmb.2009.01.010

[23] Ellis JA, Shapiro SZ, ole Moi-Yoi O, Moloo SK (1986) Lesions and saliva-specific antibody responses in rabbits with immediate and delayed hypersensitivity reactions to the bites of *Glossina morsitans centralis* . Vet Pathol 23: 661–667. 3811131 10.1177/030098588602300603

[24] Stevens WJ, Van den Abbeele J, Bridts CH (1996) Anaphylactic reaction after bites by *Glossina morsitans* (tsetse fly) in a laboratory worker. J Allergy Clin Immunol 98: 700–701. 8828549 10.1016/s0091-6749(96)70105-7

[25] Assana E, Gauci CG, Kyngdon CT, Zoli AP, Dorny P, et al (2010) Antibody responses to the host-protective *Taenia solium* oncosphere protein TSOL18 in pigs are directed against conformational epitopes. Parasite Immunol 32: 399–405. 20500670 10.1111/j.1365-3024.2009.01197.x PMC2881308

[26] Wang X, Ribeiro JM, Broce AB, Wilkerson MJ, Kanost MR (2009) An insight into the transcriptome and proteome of the salivary gland of the stable fly, *Stomoxys calcitrans* . Insect Biochem Mol Biol 39: 607–614. 19576987 10.1016/j.ibmb.2009.06.004 PMC2737351

[27] Lindh JM, Torr SJ, Vale GA, Lehane MJ (2009) Improving the cost-effectiveness of artificial visual baits for controlling the tsetse fly *Glossina fuscipes fuscipes* . PLoS Negl Trop Dis 3: e474. 19582138 10.1371/journal.pntd.0000474 PMC2699553

[28] Schwartz BS, Ribeiro JM, Goldstein MD (1990) Anti-tick antibodies: an epidemiologic tool in Lyme disease research. Am J Epidemiol 132: 58–66. 2356814 10.1093/oxfordjournals.aje.a115643

[29] Drame PM, Poinsignon A, Besnard P, Cornelie S, Le Mire J, et al (2010) Human antibody responses to the *Anopheles* salivary gSG6-P1 peptide: a novel tool for evaluating the efficacy of ITNs in malaria vector control. PLoS One 5: e15596. 21179476 10.1371/journal.pone.0015596 PMC3001874

[30] Schwarz A, Sternberg JM, Johnston V, Medrano-Mercado N, Anderson JM, et al (2009) Antibody responses of domestic animals to salivary antigens of *Triatoma infestans* as biomarkers for low-level infestation of triatomines. Int J Parasitol 39: 1021–1029. 19248784 10.1016/j.ijpara.2009.01.010 PMC2748746

[31] Volf P, Grubhoffer L, Hosek P (1993) Characterisation of salivary gland antigens of *Triatoma infestans* and antigen-specific serum antibody response in mice exposed to bites of *T. infestans* . Vet Parasitol 47: 327–337. 8333137 10.1016/0304-4017(93)90033-j

[32] Remoue F, Cisse B, Ba F, Sokhna C, Herve JP, et al (2006) Evaluation of the antibody response to *Anopheles* salivary antigens as a potential marker of risk of malaria. Trans R Soc Trop Med Hyg 100: 363–370. 16310235 10.1016/j.trstmh.2005.06.032

[33] Rohousova I, Ozensoy S, Ozbel Y, Volf P (2005) Detection of species-specific antibody response of humans and mice bitten by sand flies. Parasitology 130: 493–499. 15991492 10.1017/s003118200400681x

[34] Gidwani K, Picado A, Rijal S, Singh SP, Roy L, et al (2011) Serological markers of sand fly exposure to evaluate insecticidal nets against visceral leishmaniasis in India and Nepal: a cluster-randomized trial. PLoS Negl Trop Dis 5: e1296. 21931871 10.1371/journal.pntd.0001296 PMC3172194

[35] Vinhas V, Andrade BB, Paes F, Bomura A, Clarencio J, et al (2007) Human anti-saliva immune response following experimental exposure to the visceral leishmaniasis vector, *Lutzomyia longipalpis* . Eur J Immunol 37: 3111–3121. 17935072 10.1002/eji.200737431

[36] Orlandi-Pradines E, Almeras L, Denis de Senneville L, Barbe S, Remoue F, et al (2007) Antibody response against saliva antigens of *Anopheles gambiae* and *Aedes aegypti* in travellers in tropical Africa. Microbes Infect 9: 1454–1462. 17913537 10.1016/j.micinf.2007.07.012

[37] Drame PM, Poinsignon A, Besnard P, Le Mire J, Dos-Santos MA, et al (2010) Human antibody response to *Anopheles gambiae* saliva: an immuno-epidemiological biomarker to evaluate the efficacy of insecticide-treated nets in malaria vector control. Am J Trop Med Hyg 83: 115–121. 20595489 10.4269/ajtmh.2010.09-0684 PMC2912587

[38] Doucoure S, Mouchet F, Cornelie S, DeHecq JS, Rutee AH, et al (2012) Evaluation of the human IgG antibody response to *Aedes albopictus* saliva as a new specific biomarker of exposure to vector bites. PLoS Negl Trop Dis 6: e1487. 22363823 10.1371/journal.pntd.0001487 PMC3283547

[39] Poinsignon A, Cornelie S, Ba F, Boulanger D, Sow C, et al (2009) Human IgG response to a salivary peptide, gSG6-P1, as a new immuno-epidemiological tool for evaluating low-level exposure to *Anopheles* bites. Malar J 8: 198. 19674487 10.1186/1475-2875-8-198 PMC2733152

[40] Sagna AB, Sarr JB, Gaayeb L, Drame PM, Ndiath MO, et al (2013) gSG6-P1 salivary biomarker discriminates micro-geographical heterogeneity of human exposure to *Anopheles* bites in low and seasonal malaria areas. Parasit Vectors 6: 68. 23497646 10.1186/1756-3305-6-68 PMC3631127

[41] Schwarz A, Juarez JA, Richards J, Rath B, Machaca VQ, et al (2011) Anti-triatomine saliva immunoassays for the evaluation of impregnated netting trials against Chagas disease transmission. Int J Parasitol 41: 591–594. 21426907 10.1016/j.ijpara.2011.02.001 PMC3118394

[42] Poinsignon A, Cornelie S, Mestres-Simon M, Lanfrancotti A, Rossignol M, et al (2008) Novel peptide marker corresponding to salivary protein gSG6 potentially identifies exposure to *Anopheles* bites. PLoS One 3: e2472. 18575604 10.1371/journal.pone.0002472 PMC2427200

[43] Elanga Ndille E, Doucoure S, Damien G, Mouchet F, Drame PM, et al (2012) First attempt to validate human IgG antibody response to Nterm-34 kDa salivary peptide as biomarker for evaluating exposure to *Aedes aegypti* bites. PLoS Negl Trop Dis 6: e1905. 23166852 10.1371/journal.pntd.0001905 PMC3499371

[44] Ali ZM, Bakli M, Fontaine A, Bakkali N, Vu Hai V, et al (2012) Assessment of *Anopheles* salivary antigens as individual exposure biomarkers to species-specific malaria vector bites. Malar J 11: 439. 23276246 10.1186/1475-2875-11-439 PMC3547717

[45] King JG, Vernick KD, Hillyer JF (2011) Members of the salivary gland surface protein (SGS) family are major immunogenic components of mosquito saliva. J Biol Chem 286: 40824–40834. 21965675 10.1074/jbc.M111.280552 PMC3220476

[46] Vlkova M, Rohousova I, Hostomska J, Pohankova L, Zidkova L, et al (2012) Kinetics of antibody response in BALB/c and C57BL/6 mice bitten by *Phlebotomus papatasi* . PLoS Negl Trop Dis 6: e1719. 22802977 10.1371/journal.pntd.0001719 PMC3393673

[47] Teixeira C, Gomes R, Collin N, Reynoso D, Jochim R, et al (2010) Discovery of markers of exposure specific to bites of *Lutzomyia longipalpis*, the vector of *Leishmania infantum* chagasi in Latin America. PLoS Negl Trop Dis 4: e638. 20351786 10.1371/journal.pntd.0000638 PMC2843637

[48] Souza AP, Andrade BB, Aquino D, Entringer P, Miranda JC, et al (2010) Using recombinant proteins from *Lutzomyia longipalpis* saliva to estimate human vector exposure in visceral Leishmaniasis endemic areas. PLoS Negl Trop Dis 4: e649. 20351785 10.1371/journal.pntd.0000649 PMC2843636

[49] Sanders ML, Jaworski DC, Sanchez JL, DeFraites RF, Glass GE, et al (1998) Antibody to a cDNA-derived calreticulin protein from *Amblyomma americanum* as a biomarker of tick exposure in humans. Am J Trop Med Hyg 59: 279–285. 9715947 10.4269/ajtmh.1998.59.279

[50] Van Den Abbeele J, Caljon G, Dierick JF, Moens L, De Ridder K, et al (2007) The *Glossina morsitans* tsetse fly saliva: general characteristics and identification of novel salivary proteins. Insect Biochem Mol Biol 37: 1075–1085. 17785195 10.1016/j.ibmb.2007.06.006

[51] Maclean L, Odiit M, Sternberg JM (2006) Intrathecal cytokine responses in *Trypanosoma brucei rhodesiense* sleeping sickness patients. Trans R Soc Trop Med Hyg 100: 270–275. 16343570 10.1016/j.trstmh.2005.03.013

[52] Aksoy S, Caccone A, Galvani AP, Okedi LM (2013) *Glossina fuscipes* populations provide insights for human African trypanosomiasis transmission in Uganda. Trends Parasitol 29: 394–406. 23845311 10.1016/j.pt.2013.06.005 PMC3772539

