# Supplementary figures and images for: Serological Responses and Biomarker Evaluation in Mice and Pigs Exposed to Tsetse Fly Bites
Source: PLoS Negl Trop Dis. 2014 May 22;8(5):e2911. doi: 10.1371/journal.pntd.0002911 (PMC4031185; doi:10.1371/journal.pntd.0002911)

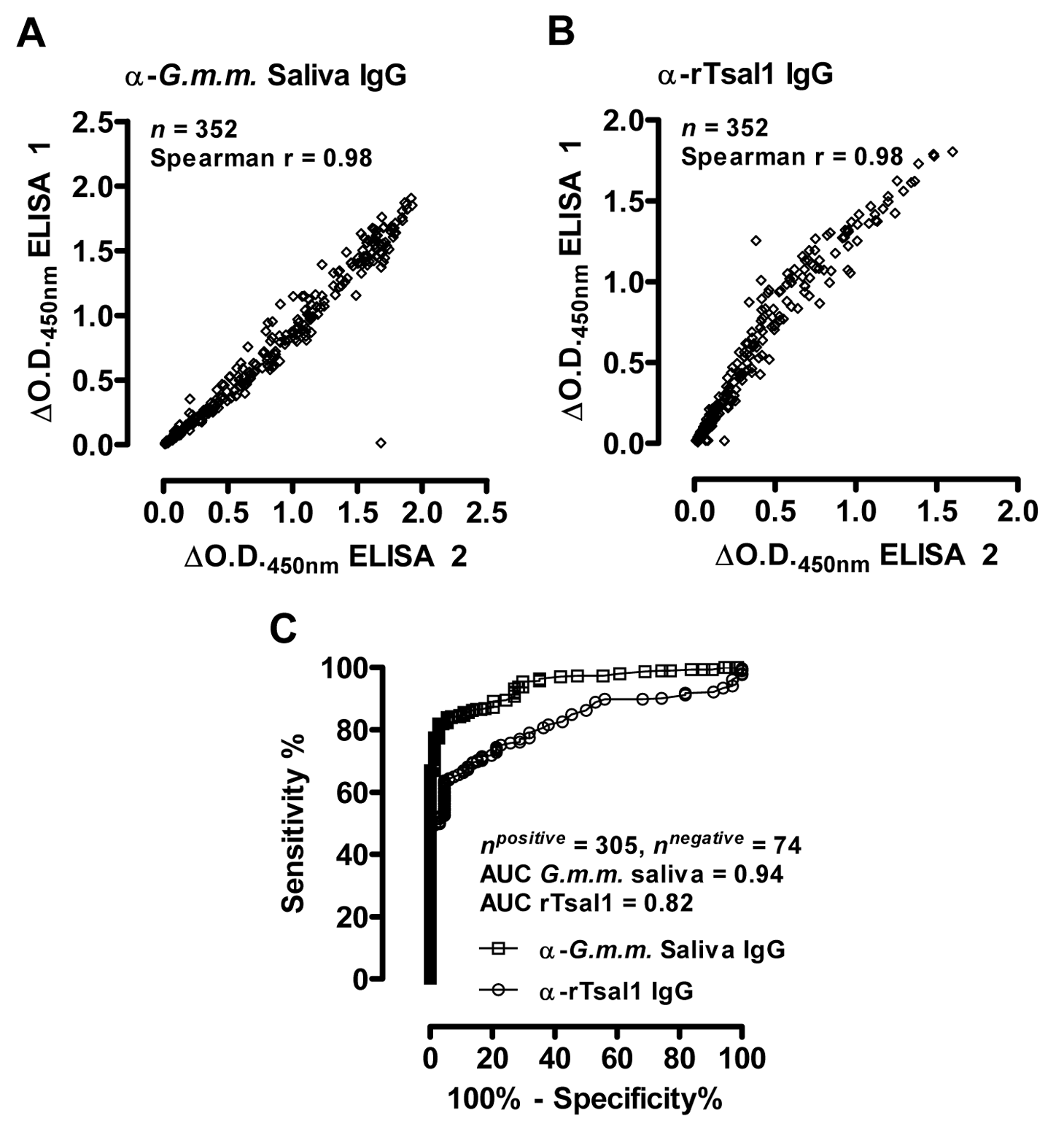

Supplement: Figure S1 — Repeatability and specificity/sensitivity analysis of the antibody detection test in mice. Scatter plot analysis of the anti-G. m. morsitans saliva IgG responses (A) and the anti-rTsal1 IgG repsonses (B) (ΔO.D.450 nm) in two separate tests performed on a panel of 1∶1600 diluted mouse plasma samples (n = 352). Test repeatability was analyzed by the non-parametric Spearman correlation test. Sensitivity and specificity of the two assays were assessed by receiver operating characteristic (ROC) curve analysis of the ΔO.D. values of exposed and non-exposed mice (C). The area under the ROC curve (AUC) is reported as a measure for the test performance. (TIF) [file pntd.0002911.s001.tif]

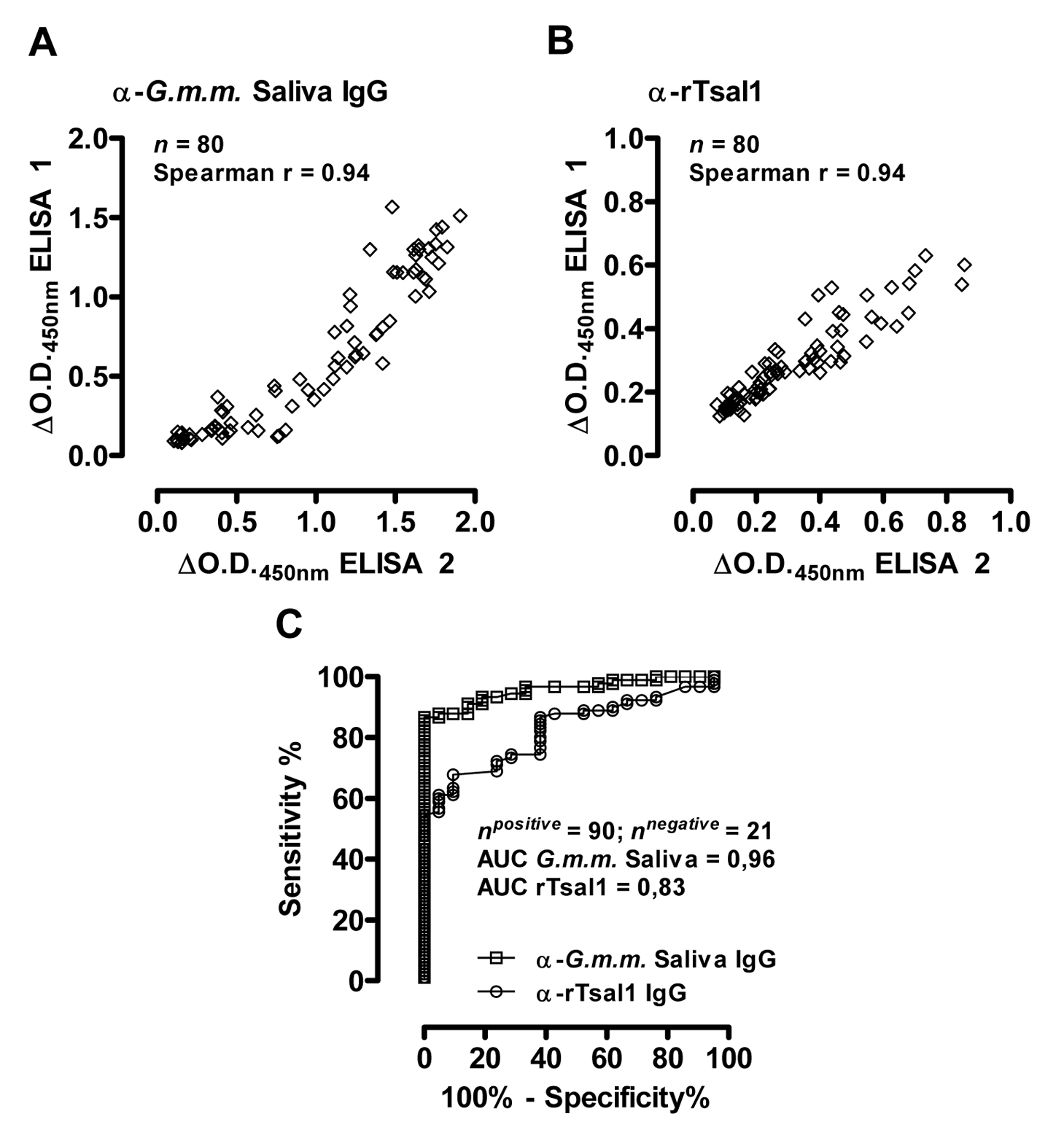

Supplement: Figure S2 — Repeatability and specificity/sensitivity analysis of the antibody detection test in pigs. Scatter plot analysis of the anti-G. m. morsitans saliva IgG responses (A) and the anti-rTsal1 IgG repsonses (B) (ΔO.D.450 nm) in two separate tests performed on a panel of 1∶1600 diluted porcine plasma samples (n = 80). Test repeatability was analyzed by the non-parametric Spearman correlation test. Sensitivity and specificity of the two assays were assessed by receiver operating characteristic (ROC) curve analysis of the ΔO.D. values of exposed and non-exposed mice (C). The area under the ROC curve (AUC) is reported as a measure for the test performance. (TIF) [file pntd.0002911.s002.tif]
